# Nutritional Evaluation of Australian Microalgae as Potential Human Health Supplements

**DOI:** 10.1371/journal.pone.0118985

**Published:** 2015-02-27

**Authors:** Megan Kent, Heather M. Welladsen, Arnold Mangott, Yan Li

**Affiliations:** 1 College of Marine and Environmental Science, James Cook University, Townsville, Australia; 2 MBD Energy Ltd, Melbourne, Australia; ICGEB, INDIA

## Abstract

This study investigated the biochemical suitability of Australian native microalgal species *Scenedesmus* sp., *Nannochloropsis* sp., *Dunaliella* sp., and a chlorophytic polyculture as nutritional supplements for human health. The four microalgal cultures were harvested during exponential growth, lyophilized, and analysed for proximate composition (moisture, ash, lipid, carbohydrates, and protein), pigments, and amino acid and fatty acid profiles. The resulting nutritional value, based on biochemical composition, was compared to commercial *Spirulina* and *Chlorella* products. The Australian native microalgae exhibited similar, and in several cases superior, organic nutritional properties relative to the assessed commercial products, with biochemical profiles rich in high-quality protein, nutritious polyunsaturated fats (such as α-linolenic acid, arachidonic acid, and eicosapentaenoic acid), and antioxidant pigments. These findings indicate that the microalgae assessed have great potential as multi-nutrient human health supplements.

## Introduction

There is well-founded, enormous potential for a greater variety of microalgal species to be utilized in human nutrition. A myriad of microalgae contain protein of high quality for humans [1]. Several of the most common microalgal pigments (chlorophyll, *β*-carotene, and lutein) are beneficial to human health, possessing cancer prevention activity and functioning as antioxidants and anti-inflammatories [2]. Nutritious polyunsaturated fatty acids also proliferate in many algal species, providing essential fatty acids necessary for cardiovascular, ocular, and neurological health [3]. Despite this potential, few microalgal species are currently utilized in the human nutrition market.

*Spirulina* and *Chlorella* currently dominate this lucrative market, and are sold in over 20 countries worldwide [3]. Commonly sought nutraceuticals from these species include protein, vitamins, pigments chlorophyll and *β*-carotene, and minerals. *Spirulina* in particular is marketed for its protein, γ-linolenic acid (GLA) and phycocyanin content. *Chlorella*, on the other hand, is advertised to provide the Chlorella Growth Factor (CGF), a water-soluble extract composed of a variety of substances including essential amino acids, peptides, proteins, vitamins, sugars, and nucleic acids [4].

Clinical and animal trials have revealed a variety of health benefits derived from *Chlorella* and *Spirulina* consumption. *Chlorella* and its extracts containing the CGF are capable of promoting growth rate, increasing insulin sensitivity, strengthening immune system function, and preventing stress-induced ulcers and pregnancy-associated anemia and hypertension [5]. The exact mechanisms behind these *Chlorella* health benefits are not well understood, but have been attributed to a combined effect of multiple nutrients [6]. *Spirulina* can improve hemoglobin, protein, and vitamin levels in malnourished children, alleviate vitamin-A deficiency through provision of bioavailable *β*-carotene, and favourably affect antioxidant capacity, immune function, and anemia status [7]. Positive health influences of Cyanobacteria, including *Spirulina*, have been attributed to fiber components, phycocyanin, γ-linolenic acid, vitamins, phenolic compounds, and minerals [8]. The resulting health benefits from these studies have provided the means to successfully market these microalgae to the public as multi-nutrient supplements.

The microalgae *Nannochloropsis* sp., *Scenedesmus* sp., and *Dunaliella* sp. are utilized for large-scale applications in the biofuel and aquaculture industries, but *Nannochloropsis* sp. and *Scenedesmus* sp. are not widely produced for human health. Although global production of *Dunaliella* sp. for human nutrition is on the rise, current estimates of microalgae production show that *Spirulina* and *Chlorella* exceed *Dunaliella* in ton/year production by ten and four-fold, respectively [9]. Productivity, growth media requirements, environmental tolerances, and other culturing data are already established for *Nannochloropsis* sp., *Scenedesmus* sp., and *Dunaliella* sp., creating the potential for efficient production, or expansion in production, of nutraceutical biomass. Australia, with its abundant sunshine and non-arable land, is a suitable prospective location for cultivation [10].

Current findings highlight individual nutritious biochemical components of *Nannochloropsis* sp., *Scenedesmus* sp., and *Dunaliella* sp., and initial food acceptability, preparation, and animal and human trials indicate that they may have an opportune future in health food applications. *Nannochloropsis* sp., and *Scenedesmus* sp., have previously been suggested for nutraceutical application due to their concentrations of the nutritious fatty acid eicosapentaenoic acid (EPA), and vitamins and essential minerals, respectively [11,12]. *Dunaliella* sp. is principally highlighted in nutrition for its ability to accumulate high concentrations of carotenes and xanthophylls [5]. Acceptability studies utilizing *Scenedesmus* sp. as a protein supplement for humans have yielded principally positive results [13]. In rat trials, *Scenedesmus acutus* significantly improved growth in wheat and bread diets when utilized as a protein supplement [14], and has been shown to contain comparable protein efficiency ratios, biological values (measure of nitrogen retained for growth and maintenance), and digestibility coefficients (measures protein quality) to *Spirulina* and *Chlorella* [1]. *Nannochloropsis* sp. and *Dunaliella bardawil* supplementation in rats was innocuous when fed in quantities as high as 10% of their diet [11,15]. Other rat nutrition studies have shown that plasma cholesterol, triglycerides and creatine phosphokinase levels could be significantly lowered when *Dunaliella tertiolecta* was used as the sole protein source for just 12 days [16]. Studies have highlighted the capacity for *β*-carotene from *Dunaliella* species to act as an antioxidant or anti-hyperlipidemic, and for EPA from *Nannochloropsis oculata* to elevate EPA level in human blood, but no known studies have tested other nutritious components of either algae in human trials [17].

Methods for breaking *Scenedesmus obliquus* and *Scenedesmus quadricauda* cell walls, drying biomass, and food preparation in order to increase digestibility and nutrient availability have already been determined, much like commercially-produced *Chlorella vulgaris* [18,19]. Furthermore, *Scenedesmus* sp. and *Dunaliella* sp. have attractive taste similar to *Chlorella vulgaris*, and would do well when incorporated into many foods, such as pasta, pretzels, potato and corn chips, soups and seasonings, an assortment of dairy products, and even candies and ice creams [13].

Microalgal polycultures, especially photoautotrophic mixes, are commonly utilized in aquaculture systems as a source of nutrition for fish and crustaceans, and in bioremediation [20,21]. To our knowledge they have yet to be cultivated for human nutrition, however, supplements are currently sold which combine several microalgal species in one multi-nutrient supplement. The ability to culture a microalgal polyculture for nutritional applications may provide several advantages over monoculture production, including increased productivity and robustness to predation by contaminants such as rotifers due to variation in cell size and structure [22].

Although individual nutrients (such as specific fatty acids or antioxidant pigments) have been highlighted as nutritious components in *Nannochloropsis* sp., *Scenedesmus* sp., and *Dunaliella* sp., and initial focused animal and human trials are promising, it is unclear whether these microalgae are suitable for human nutrition in a multi-nutrient capacity like commercially produced *Spirulina* and *Chlorella*. Furthermore, no knowledge is currently available regarding whether microalgal polycultures could also merit consideration in human nutrition applications.

Although biochemical composition data may be available for *Spirulina* and *Chlorella* products, it is essential to re-assess their composition for an accurate comparison to the microalgae tested in this work since differing methods utilized for biochemical profiling in nutrition labelling and scientific publication, especially in the cases of protein and carbohydrate content(s), are known to yield variable results [23,24]. This study aimed to determine the biochemical suitability of Australian microalgae, *Scenedesmus* sp., *Nannochloropsis* sp., and *Dunaliella* sp., plus a unique chlorophytic polyculture (CPC) for human nutrition through examination of biochemical profiles and comparison to existing commercially available *Chlorella* and *Spirulina* products.

## Materials and Methods

### Microalgae growth and sample preparation

This study was conducted at the James Cook University/MBD Energy (JCU/MBD) Microalgae Research and Development Facility (146°45&rsquo;38"E, 19°19&rsquo;39"S). Microalgae *Scenedesmus* sp., *Nannochloropsis* sp., *Dunaliella* sp., and a designed freshwater chlorophytic polyculture (CPC; consisting of *Schroederiella apiculata*, *Scenedesmus pectinatus*, *Tetraedrom minimum*, *Mesotaenium* sp. and *Desmodesmus sp*.) were isolated from various locations in Australia (no specific collection permissions were required) (Table 1) and DNA-sequenced by Macrogen (Seoul, Korea). These microalgae were batch-cultured in three replicate vertical, conical-based fibreglass (40cm diameter, 3m height, 320L volume) photobioreactors with central aeration, utilizing media as depicted in Table 1. Culture pH was maintained at 6.5–7.5 and 7.5–8.5 by injection of CO_2_ for 2 ppt and 36 ppt cultures, respectively. Microalgal cultures were grown at a mean temperature of 25°C, under a light:dark regime of 18:6 hours and a photon flux density of 80 μmol m^2^ s^-1^. Growth was established for each algal culture using cell counts with a Neubauer hemocytometer and gravimetric dry weight determination. All cultures were harvested by centrifugation at exponential growth phase to optimize protein and highly-unsaturated fatty acid content. Post-harvest, the microalgal biomass was lyophilized and kept in air-tight containers in darkness at -80°C for subsequent biochemical profiling analyses. The drying method of choice for commercial producers of high-value microalgae products such as *Spirulina* and *Chlorella* is spray-drying [25], however, lyophilized microalgal biomass has been found to be similar to spray-dried biomass in elemental composition and contents of protein, carbohydrates, chlorophylls, and fatty acids [26]- therefore drying method was not a variable for the purpose of this study.

**Table 1:** Culturing parameters for each James Cook University/MBD Energy (JCU/MBD) microalgae.

	*Nannochloropsis* sp.	*Scenedesmus* sp.	*Dunaliella* sp.	CPC^*a*^
**Media**	f/2 [27]	Bold Basal Medium [28]	Modified Johnson Media [29]	Bold Basal Medium [28]
**Salinity**	36ppt	2ppt	36ppt	2ppt
**Australian isolate origin**	Great Barrier Reef	Tarong Power Station, Queensland	Pink Lake; Goldsfields-Esperance region, Western Australia	Tarong Power Station, Queensland

^*a*^ Chlorophytic polyculture (CPC) species—*Schroederiella apiculata*, *Scenedesmus pectinatus*, *Tetraedrom minimum*, *Mesotaenium* sp. and *Desmodesmus* sp.- were isolated from the same location.

Four *Spirulina* (from producers in China, India, Taiwan, and the USA) and three *Chlorella* (from producers in Japan, South Korea, and Germany) supplement powders containing only 100% pure *Spirulina platensis* or *Chlorella vulgaris*, respectively, were purchased in May 2013, to act as references for commercial microalgal products. These commercial products were kept unopened in darkness at 20°C until commencement of biochemical profiling. Once products were opened, an aliquot of the product was maintained in darkness in an airtight container at -80°C for biochemical profiling. For biochemical quantification and statistical analysis the four *Spirulina* and three *Chlorella* products were treated as replicates.

### Biochemical composition

**Fatty acid (FA) analysis**. FA analyses were performed in duplicate for each replicate sample, and biochemical results are presented as mean values. FAs were simultaneously extracted and esterified in a direct transesterification method, as described in detail in Gosch et al. [30]. The resulting fatty acid methyl esters (FAMEs) were separated and quantified on an Agilent 7890 GC (DB-23 capillary column, 60 m x 0.25 mm I.D. x 0.15 μm) and an Agilent 5975C Electron Ionisation (EI) Turbo Mass Spectrometer (Agilent Technologies Australia Pty Ltd). The column temperature gradient was programmed following David et al. [31], ramping from 50°C to 250°C. The quantity and identity of fatty acids were determined using external standards (Sigma Aldrich) and NIST08 Mass Spectral Library, and corrected for recovery of internal standard (C19:0). Total FA content was determined as the sum of all FAMEs.

**Pigment analysis**. Pigments were assessed via methods adapted from Van Heukelem and Thomas [32] utilizing the studies’ top-performing column, running solvents and column flow rate, but with modified extraction and gradient system. All pigment extraction procedure steps were performed on ice in dimmed light. Twenty mg of each sample was extracted with methanol: 0.5 M tert-Butyl acetoacetate (TBAA) (99:1 v/v) using mechanical disruption via zirconium beads agitated by a bullet blender (model BBY24M). After bead disruption, samples were centrifuged (Sigma 1–14 Microfuge) at 13000 rpm for 5 minutes, and supernatant was removed. A cycle of bead disruption, centrifugation, and supernatant removal was repeated until sample pellet and supernatant were colourless. An aliquot of the combined pigment extract was then diluted by 71% with 28 mM TBAA (pH 6.5) and 100 *μ*L of the solution was injected onto a 60°C Agilent Eclipse XDB C-8 (4.6 x 150 mm; 3.5 *μ*m) column on a Varian Prostar HPLC with Varian Prostar UV-Viz detector and Metachem Degassit degasser unit at a flow rate of 1 ml min^-1^. The HPLC solvent system was set as follows: 5% methanol (100%) (solvent B): 95% methanol and 28 mM TBAA pH 6.5 70:30 (v/v) (solvent A) for 0–15 minutes; 50% solvent B and 50% solvent A for 15–38 minutes; 95% solvent B and 5% solvent A for 38–45 minutes; 100% solvent B for 45–47 minutes; 5% solvent B and 95% solvent A for 47–52 minutes. Pigments were detected at 440 nm and identified by comparison with retention times of phytoplankton standards (DHI Laboratory Products, Denmark). In this work the chlorophyll summation was defined as the sum of chlorophylls *a* and *b*, while the carotenoid summation was defined as the sum of astaxanthin, lutein, zeaxanthin, echinenone, and *β*-carotene. Astaxanthin and *β*-carotene results were also reported individually since these carotenoids are of particular interest for health food applications due to their well-known properties as antioxidants [33].

**Amino acid analysis**. Equal portions of each replicate were pooled to form one amalgamated sample of each microalgal species for amino acid analysis. Amino acid profile of each sample was analysed by the Instrument Analysis Center of Shanghai Jiao Tong University, utilizing a Hitachi L8900 Amino Acid Analyzer (Tokyo, Japan) according to the Chinese national standard protocol for amino acid determination (GB/T 5009.124–2003). Briefly, 10 mg of each sample was hydrolysed in a vacuum glass tube using 6 M HCl at 110°C for 22h. Samples were then dried in a vacuum, and the residue was diluted with 0.02 M HCl prior to analysis on the amino acid analyser, together with amino acid standards. In this work, protein was measured in the method currently recommended by the FAO- the sum of amino acids [34]- excluding tryptophan.

**Essential amino acid index (EAAI)**. EAAI scores were calculated to indicate protein quality as in Tabarsa et al. [35] using a FAO/WHO established human reference pattern [36]. EAAI scores represented protein quality by comparing ratios of essential amino acids in a food item to those of a reference pattern derived from whole body tissue of the animal [36]. In EAAI computation, index scores approaching 1 represented the closest possible match between a food’s essential amino acid profile and the consumer. In the evaluation, a score of ~ > 0.95 defined a ‘high’ quality protein, while a score of ~ 0.86–0.95 signified a ‘good’ quality protein, a score of ~ 0.75–0.86 signified a ‘useful’ protein, and a score of ~ ≤ 0.75 indicated an ‘inadequate’ protein [37].

**Proximate analysis**. Ash, moisture, crude protein, crude fat, and a measure representing sugars and starches (carbohydrates) were analysed to determine proximate composition; crude fiber was not assessed as it is not recommended for inclusion in food composition databases [34]. The proximate system of analysis is commonly utilized for providing a broad classification of food components in nutrition and forms the basis for legislative regulation of food analysis in many countries [34]. Proximate analyses were performed in duplicate for each sample, and biochemical values are presented as means in the results. Carbohydrates were analyzed utilizing a colorimetric technique approved by the FAO [34]- the phenol-sulphuric acid spectrophotometric method. Lipids were extracted from samples via direct extraction by methods modified from Lewis et al. [38] to use less toxic solvents (methanol and hexane), as in Carvalho and Malcata [39]. Total lipid extraction recovered neutral lipids, phospholipids, glycolipids, and pigments. Moisture and ash content were determined gravimetrically after algal biomass was exposed to 100°C in a Qualtex Thermostat z670 drying oven for 2 hours, and 500°C in a Yokogawa model UP150 muffle furnace for 8 hours, respectively.

### Statistical Analysis

All statistical analyses on individual biochemical parameter data were carried out using IBM SPSS Statistics 20. The assumptions of homogeneity of variance and normality were assessed by scatter plots of residuals and normal curves of residuals, respectively. Proportional data were arcsine-square root transformed to meet ANOVA assumptions. One-way ANOVAs with Tukey HSD post-hocs were utilized to discern differences between groups. Principle Component Analysis (PCA) was conducted using Primer 6 on all biochemical parameter data (excepting amino acid data due to pooled replicates) to better understand how individual parameters drove differences between algal samples. The data for PCA was normalized on a common measurement scale. A subsequent permutational multivariate analysis of variance (PERMANOVA) and pair-wise comparisons were used to detect significant differences between samples. Differences were deemed significant at *p* < 0.05.

## Results and Discussion

**Fig 1:**
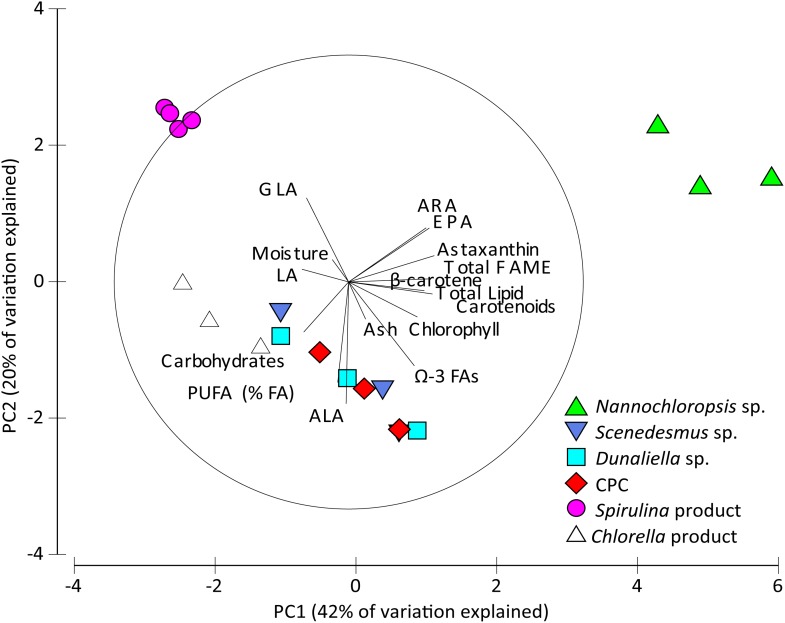
Principal component analysis (PCA) depicting extent of differences in biochemical parameters between James Cook University/MBD Energy (JCU/MBD) microalgae (*Nannochloropsis* sp., *Dunaliella* sp., *Scenedesmus* sp., and the CPC) and commercial microalgae products, which are indicated by the length of radiating lines. Biochemical parameters corresponding to the radiating lines pointing towards species indicate parameters of similarity, while opposite radiating lines indicate dissimilarity.

PCA analysis demonstrated biochemical similarity through clustering between *Chlorella* products, *Dunaliella* sp., CPC, and *Scenedesmus* sp. *Spirulina* products (PERMANOVA; *p* < 0.05) and *Nannochloropsis* sp. (PERMANOVA; *p* > 0.05) were divergent from all other microalgae. These divergences were driven by GLA and Ω-3 FA content (lack of) in *Spirulina* and high EPA, arachidonic acid (ARA), and astaxanthin content in *Nannochloropsis* sp. (Fig. 1). PERMANOVA analysis of biochemical values also demonstrated similarities between all JCU/MBD microalgae and *Chlorella* products (*p* > 0.05). These findings indicate that the JCU/MBD microalgae presented here- *Dunaliella* sp., *Nannochloropsis* sp., *Scenedesmus* sp., and the CPC- exhibited comparable, and in several cases superior, organic nutritional properties relative to *Spirulina* or *Chlorella* commercial microalgal products.

### Fatty acids

All JCU/MBD microalgae were superior to one or both commercial microalgal products in terms of total Ω-3 FA content: *Nannochloropsis* sp. and the CPC contained significantly greater Ω-3 FA content than *Chlorella* products (*p* < 0.05; Table 2), and all JCU/MBD microalgae contained significantly greater Ω-3 FA content than *Spirulina* products, which contained no detectable Ω-3 FAs (Table 2).

**Table 2:** Mean fatty acid (FA) content (mg g^-1^ dry weight) of nutritious FAs, proportion of polyunsaturated FAs (PUFA; % of total FAs), ratio of Ω-6/Ω-3 FAs, and FA totals (mg g^-1^ dry weight) in James Cook University/MBD Energy (JCU/MBD) microalgae and commercial microalgae products (mean ± SD, n = 3).

	JCU/MBD Microalgae				Products	
	*Nannochloropsis* sp.	*Scenedesmus* sp.	*Dunaliella* sp.	CPC	*Spirulina*	*Chlorella*
**ALA (C18:3 n-3)^*a*^**	0.23 ± 0.11^a^, ^b^	21.22 ± 6.88^b^, ^c^, ^d^	21.18 ± 4.28^b^, ^c^, ^d^	23.70 ± 2.53^c^, ^d^	ND^a^, ^b^	10.35 ± 8.24^a^, ^b^, ^c^
**ARA (C20:4 n-6)^*b*^**	7.45 ± 1.79^b^	ND^a^	ND^a^	0.41 ± 0.09^a^	ND^a^	ND^a^
**EPA (C20:5 n-3)^*c*^**	36.76 ± 2.93^b^	0.11 ± 0.19^a^	ND^a^	0.51 ± 0.06^a^	ND^a^	ND^a^
**GLA (C18:3 n-6)^*d*^**	0.82 ± 0.3^a^, ^b^, ^c^	0.49 ± 0.16^a^, ^b^, ^c^	2.53 ± 0.59^b^, ^c^	0.61 ± 0.20^a^, ^b^, ^c^	9.42 ± 1.52^d^	ND^a^, ^b^
**LA (C18:2 n-6)^*e*^**	4.11 ± 1.46^a^, ^b^, ^c^	6.19 ± 2.90^a^, ^b^, ^c^, ^d^, ^e^	8.43 ± 3.72^a^, ^b^, ^c^, ^d^, ^e^	4.46 ± 1.11^a^, ^b^, ^c^, ^d^	10.62 ± 1.40^b^, ^c^, ^d^, ^e^	18.45 ± 11.80^c^, ^d^, ^e^
**PUFA (% FA)**	44.64 ± 3.35^a^, ^b^	51.14 ± 7.04^a^, ^b^, ^c^	71.67 ± 2.40^c^, ^d^	65.69 ± 1.96^b^, ^c^, ^d^	41.60 ± 1.01^a^, ^b^	70.59 ± 3.21^c^, ^d^
**Ω-6/Ω-3 Ratio**	0.35 ± 0.05	0.22 ± 0.08	0.43 ± 0.20	0.17 ± 0.02	Undefined	7.20 ± 11.16
**Total Ω-3 FA**	36.99 ± 1.75^c^, ^d^	34.21 ± 6.08^b^, ^c^, ^d^	33.05 ± 3.88^b^, ^c^, ^d^	36.70 ± 3.52^c^, ^d^	ND^a^, ^b^	15.84 ± 7.20^a^, ^b^, ^c^
**Total FA**	114.57 ± 9.02^d^	82.08 ± 7.65^b^, ^c^	66.32 ± 5.00^a^, ^b^, ^c^	66.39 ± 5.05^a^, ^b^, ^c^	48.73 ± 1.14^a^, ^b^	60.60 ± 3.26^a^, ^b^, ^c^

Values in rows with different superscripts represent significant differences between microalgal samples (p < 0.05). ND signifies ‘not detected’.

^*a*^ *α*-Linolenic acid

^*b*^ Arachidonic acid

^*c*^ Eicosapentaenoic acid

^*d*^ γ-Linolenic acid

^*e*^ Linoleic acid.

The higher Ω-3 FA content of JCU/MBD microalgae relative to commercial products was driven by elevated concentrations of nutritious, long-chain essential Ω-3 FAs. All JCU/MBD microalgae were rich (> 2% dry weight) in either EPA or α-linolenic acid (ALA) (Table 2). Humans are incapable of synthesizing ALA de novo [40], and can only inefficiently synthesize EPA from precursor ALA [41], thus provision of these fatty acids in our diet is essential. Quantities of the FAs ARA and EPA were significantly higher in *Nannochloropsis* sp. than in all other microalgae samples (*p* < 0.05; Table 2). At a mean of 3.68% (dry weight), the quantity of EPA achieved in *Nannochloropsis* sp. biomass was more than 100-fold greater than in all other samples tested. This concentration of EPA is within the range of 3.18–4.33% recently achieved by researchers aiming to produce high quantities of EPA in *Nannochloropsis* sp. [42]. The CPC, *Scenedesmus* sp., and *Dunaliella* sp. contained significantly greater quantities of essential FA ALA than either commercial product species (*p* <0.05), while *Spirulina* products contained significantly greater quantities of the Ω-6 FA, γ-linolenic acid (GLA) than all other samples (*p* < 0.05). All microalgae contained similar amounts of *cis*-linoleic acid (LA) (*p* > 0.05), apart from *Nannochloropsis* sp. which contained significantly less (*p* < 0.05; Table 2). Docosahexaenoic acid (DHA) was not detected in any of the microalgal samples.

Essential FAs (especially EPA and DHA) are known to be beneficial for human health, reducing the rate of occurrence and lowering the risk of cardiovascular and artery diseases, decreasing levels of blood cholesterol, providing for maximal brain function and visual acuity (especially in infants), and reducing inflammation and arthritis [43]. Therefore, the content of these FAs is nutritionally critical for evaluating microalgal potential for human health benefit.

PUFA content was similar between *Nannochloropsis* sp., *Scenedesmus* sp., and *Spirulina* products, ranging from 41.60–51.14% of fatty acid content (*p* > 0.05). *Dunaliella* sp., the CPC, and *Chlorella* products contained greater PUFA content representing 65.69–71.67% of fatty acids (*p* > 0.05). All JCU/MBD microalgae were comparable in total FA content to commercial products (*p* > 0.05) except for *Nannochloropsis*, which contained significantly greater total fatty acid content (*p* < 0.05; Table 2).

Ω-6/Ω-3 ratios in all JCU/MBD microalgae were within the range recommended as most beneficial for human health, 0.25–1 (Table 2) [44]. Ratios in commercial products, however, exceeded recommended levels, with highly variable *Chlorella* ratios (averaging 7.2) and *Spirulina* ratios as undefined, since no Ω-3 FAs were present to balance the 20.04 ±1.97 mg g^-1^ of Ω-6s in *Spirulina* samples. Due to competition for desaturation enzymes, a balance between Ω-6 and Ω-3 FA in foods is essential for human health benefits to be conveyed, and high Ω-6/Ω-3 ratios have been implicated in the promotion of cardiovascular disease, cancer, and inflammatory and autoimmune diseases [44]. This signifies that in addition to having high essential fatty acid content, JCU/MBD microalgae assessed were superior to the commercial products assessed in terms of Ω-6/Ω-3 balance.

### Pigments

*Nannochloropsis* sp. and *Dunaliella* sp. had a significantly greater sum of carotenoids than both commercial products (*p* < 0.05), whereas other JCU/MBD microalgae were similar in carotenoid content to products (*p* > 0.05; Table 3). *Nannochloropsis* sp. also contained significantly greater quantities of the nutritious carotenoid astaxanthin than all microalgae sampled (*p* < 0.05, Table 3). At a level of 0.64% dry weight, astaxanthin content of *Nannochloropsis* sp. found in this study nears the level of 0.7% achieved by Lubian et al. [45], supporting the conclusion that *Nannochloropsis* could be a source of valuable pigments due to its ability to achieve high production levels of a range of pigments- including astaxanthin. All microalgae samples contained equivalent minimal *β*-carotene content (*p* > 0.05, Table 3). Although JCU/MBD microalgae contained higher mean chlorophyll content than commercial products, the differences were not significant (*p* > 0.05; Table 3). Nevertheless, the JCU/MBD microalgae presented here, especially *Nannochloropsis* sp., proved to be rich sources of chlorophyll, astaxanthin, and carotenoids, all widely recognized for their antioxidant and antimutagenic properties [2].

**Table 3:** Mean pigment content (mg g^-1^ dry weight) of James Cook University/MBD Energy (JCU/MBD) microalgae and commercial microalgae products (mean ± SD, n = 3).

	JCU/MBD Microalgae				Products	
	*Nannochloropsis* sp.	*Scenedesmus* sp.	*Dunaliella* sp.	CPC	*Spirulina*	*Chlorella*
**Chlorophyll summation**	30.54 ± 4.36^a^	19.00 ± 7.13^a^	23.65 ± 8.68^a^	24.97 ± 4.25^a^	12.33 ± 1.09^a^	8.58 ± 0.33^a^
**Carotenoid summation**	8.57 ± 1.56^c, d^	4.23 ± 0.81^a, b, c^	5.12 ± 0.72^b, c, d^	3.82 ± 0.42^a, b, c^	1.45 ± 0.38^a, b^	1.17 ± 0.44^a, b^
**Astaxanthin**	6.40 ± 1.20^b^	1.50 ± 0.32^a^	0.83 ± 0.33^a^	0.75 ± 0.22^a^	ND^a^	0.08 ± 0.03^a^
**β-carotene**	0.67 ± 0.15^a^	0.70 ± 0.13^a^	1.02 ± 0.28^a^	0.73 ± 0.09^a^	0.92 ± 0.30^a^	0.19 ± 0.09^a^

Values in rows with different superscripts represent significant differences between microalgal samples (p < 0.05). ND signifies ‘not detected’.

### Protein content and quality

Amalgamated protein content was highest in *Spirulina* products, followed by *Chlorella* products, the CPC, *Dunaliella* sp., *Scenedesmus* sp., and *Nannochloropsis* sp. (Table 4). Nevertheless, with protein content ranging from 30–52%, all JCU/MBD microalgae evaluated, as well as the commercial products, exceeded the 20% (dry weight) protein supplement baseline established by the Ontario Ministry of Agriculture and Food for animal feeds [46]. As of yet there is no established numerical baseline to define a human protein supplement.

**Table 4:** Mean (% of dry weight) proximate biochemical composition of James Cook University/MBD Energy (JCU/MBD) microalgae and commercial microalgae products (mean ± SD, *n* = 3).

	JCU/MBD Microalgae				Products	
	*Nannochloropsis* sp.	*Scenedesmus* sp.	*Dunaliella* sp.	CPC	*Spirulina*	*Chlorella*
**Ash**	11.32 ± 4.32^a^ ^, b, c^	15.72 ± 8.63^a^ ^, b, c^	19.29 ± 7.91^b, c^	7.96 ± 1.51^a^ ^, b, c^	7.02 ± 0.77^a^ ^, b^	5.71 ± 1.71^a^ ^, b^
**Carbohydrates**	9.62 ± 1.24^a^ ^, b, c^	27.66 ± 4.48^c, d, e^	14.57 ± 4.79^a^ ^, b, c, d^	18.70 ± 1.26^a^ ^, b, c, d, e^	16.00 ± 1.60^a^ ^, b, c, d^	24.93 ± 7.23^b, c, d, e^
**Total Lipid**	21.78 ± 1.71^d^	15.07 ± 1.63^b, c^	14.36 ± 2.21^a^ ^, b, c^	16.43 ± 0.40^b, c^	11.18 ± 0.37^a^ ^, b^	16.15 ± 2.06^b, c^
**Moisture**	1.84 ± 2.38^a^	0.71 ± 0.83^a^	4.05 ± 6.46^a^	2.31 ± 2.60^a^	2.59 ± 0.75^a^	1.30 ± 0.58^a^
**Protein^*a*^**	30.29	30.99	34.17	37.55	51.56	39.98

Values in rows with different superscripts represent significant differences between microalgal samples (p < 0.05).

^*a*^Protein results do not include SD since replicates were pooled for analysis.

Protein quality for human nutrition, according to EAAI scores, was ‘high’ for all four JCU/MBD microalgae (Table 5). Commercial products contained protein of slightly lower quality: protein in *Chlorella* products was classified as ‘good’ quality protein, while *Spirulina* products contained ‘useful’ protein (Table 5). EAAI scores were higher in JCU/MBD microalgae principally due to higher proportions of the essential amino acids histidine and phenylalanine. Additionally, JCU/MBD microalgae had a higher proportion of threonine than that in *Chlorella* products (*Nannochloropsis* sp. marginally), and also higher proportions of lysine and slightly higher leucine than *Spirulina* products (Table 5). Across all microalgal samples the dominant amino acid was glutamic acid and the limiting amino acid was cysteine (Table 5), a common trend in microalgae [47].

**Table 5:** Amino acid residue content (mg g^-1^ dry weight) and essential amino acid index (EAAI) value of James Cook University/MBD Energy (JCU/MBD) microalgae and commercial microalgae products.

	JCU/MBD Microalgae				Products	
	*Nannochloropsis* sp.	*Scenedesmus* sp.	*Dunaliella* sp.	CPC	*Spirulina*	*Chlorella*
Histidine*	26.26	26.06	25.03	27.79	20.07	24.32
**Serine**	42.14	45.51	43.97	45.49	48.05	40.40
**Arginine**	60.82	64.13	65.92	65.42	75.86	71.46
**Glycine**	52.12	55.98	57.04	57.15	45.58	53.84
**Aspartic acid**	91.56	101.89	105.05	104.32	101.82	93.64
**Glutamic acid**	137.82	129.37	136.11	122.80	160.59	128.88
Threonine*	48.56	56.27	50.53	53.06	51.10	47.38
**Alanine**	68.20	81.11	77.65	78.40	73.76	83.40
**Proline**	82.77	48.36	49.06	48.79	32.63	47.83
Lysine*	68.31	66.61	62.00	71.61	50.94	88.87
Tyrosine*	39.74	43.40	40.65	43.15	47.82	41.60
Methionine*	23.60	24.44	25.28	23.87	28.82	22.30
Valine*	60.24	61.76	59.83	61.59	63.64	61.02
Isoleucine*	47.22	44.10	45.08	44.24	58.37	43.98
Leucine*	94.05	91.89	93.22	92.05	90.23	92.00
Phenylalanine*	55.26	55.72	59.59	58.23	47.87	54.73
Cysteine*	*1.32*	*3.40*	*3.98*	*2.03*	*2.87*	*4.35*
**Total**	**302.95**	**309.85**	**341.68**	**375.50**	**515.65**	**399.78**
**EAAI**	1.02	1.00	0.98	1.05	0.81	0.92

Asterisks (*) indicate essential amino acids. Values in italics denote the limiting amino acid in each microalga.

### Proximate composition

Proximate composition was comparable between most JCU/MBD microalgae and commercial *Spirulina* and *Chlorella* products (Table 4).

All JCU/MBD microalgae had similar carbohydrate content to either *Spirulina* or *Chlorella* products (*p* > 0.05; Table 4). Algal carbohydrates can provide human health benefits in the form of anticoagulants, antivirals, dietary fibers, and antioxidants [48]. Carbohydrates are not sought after as a large percentage of commercial microalgal supplements, however, since large quantities of carbohydrates correspond to lower fractions of other macronutrients in the supplement, most notably protein.

All JCU/MBD microalgae, apart from *Nannochloropsis* sp., also had comparable total lipid values to one of the two commercial product species (*p* > 0.05; Table 4). *Nannochloropsis* sp. contained higher total lipid content than all commercial microalgal products (*p* < 0.05). Although the value for total lipid may be of limited nutritional significance since it assesses the quantity of a group of compounds including fatty acids, sterols, vitamins, pigments, and other lipid-solvent soluble substances, it is still widely reported and retained for food labelling and regulatory purposes [34]- and so is utilized in this work as one of the indicators of nutritional similarity between commercial microalgal products and JCU/MBD microalgae.

All microalgal samples showed a similar moisture content of below 5% (dry weight) (*p* > 0.05; Table 4). The ability to produce a microalgal powder with low moisture content is important for preservation of nutritional integrity, since presence of liquid water can encourage microbiological activity and deterioration of food products. In this regard, JCU/MBD microalgae had similar quality to *Chlorella* and *Spirulina* commercial products.

Ash content was also similar between all microalgal groups (*p* > 0.05; Table 4) except for *Dunaliella* sp., which contained greater ash content than both commercial products (*p* < 0.05, Table 4). Ash content of all microalgae tested was, nevertheless, lower than the maximum allowed in algal products sold in the USA (45% dry weight), and comparable or slightly greater than in land vegetables (5–10% dry weight) [49]. Overly high ash content in foods is generally undesirable; however, algal ash can contribute to meeting the recommended daily intake of minerals in human nutrition [50].

For future consideration of microalgal species in human nutrition, elemental and trace metal analysis would need to be undertaken to identify specific nutritious trace elements and ensure that safe limits of heavy metals are not exceeded. Elemental composition, as well as vitamin content, were not analysed in this work since mineral and vitamin content of microalgae are known to be directly related to levels of both in the microalgal growth environment [51,52], and so can be manipulated by the grower to ensure that correct levels are achieved.

### Application

*Dunaliella* sp., *Scenedesmus* sp., and (especially) *Nannochloropsis* sp., were found to have nutritious biochemical profiles, and all have the potential for conversion to, or expansion of, production for human nutrition due to their established cultivation histories in aquaculture, biofuels, or targeted nutraceuticals. This work also demonstrated that the amalgam of microalgae in the CPC (*Schroederiella apiculata*, *Scenedesmus pectinatus*, *Tetraedrom minimum*, *Mesotaenium* sp. and *Desmodesmus sp*.) was comparable in most organic nutrient aspects- and superior in ALA and Ω-3 FA content- to the commercially produced microalgal supplement products. Although the growth of microalgal polycultures may have some cultivation advantages (as previously stated), species composition and dominance have the ability to adjust in response to environmental change, which could likely affect overall biochemical composition of the biomass. Additional analyses, therefore, need to be completed to determine the degree of variability of the CPC nutritional results.

These findings warrant an expansion in exploration of the health benefits which may be possible by consuming *Dunaliella* sp., *Scenedesmus* sp., and *Nannochloropsis* sp. in whole or cracked-cell form. Like commercially produced *Spirulina* and *Chlorella*, they too may have multiple health benefits which may be conveyed by their various nutritional properties. Furthermore, utilization of whole or cracked-cell microalgae for supplement production may be more economical and environmentally sound than production of an alga for a singular nutritive compound, since it omits the large expenses and chemicals that can be needed for biorefinery-extraction and processing.

Current research also suggests that *Chlorella*, *Scenedesmus*, and *Dunaliella* are capable of producing recombinant proteins at levels similar to the model microalgae *Chlamydomonas reinhardtii*, which could lead to their use in production of antibodies, immunotoxins for use in anti-cancer therapy, growth hormones, vaccines, gut-active nutraceuticals, and therapeutic enzymes [53]. Recent innovations in genetics have yielded the potential for improvement of the already nutritious biochemical profiles of these microalgae, and highlight the further ability to utilize them as tools for manufacture and delivery of therapeutics. *Nannochloropsis* is quickly becoming a forerunner in gene knockout and replacement research, paving the way for efforts to enable enhanced production of desirable fats and other nutritious biological compounds in other ‘wild’ species [54].

## Conclusions

*Nannochloropsis* sp., *Dunaliella* sp., *Scenedesmus* sp., and the CPC exhibited exemplary biochemical profiles rich in high-quality protein, nutritious polyunsaturated fatty acids (such as ALA, ARA, and EPA), and antioxidant pigments. These properties, in addition to their comparability to commercially produced *Chlorella* and *Spirulina* products in basic biochemical composition, indicate that these microalgae have great potential for use in human health in a multi-nutrient capacity. *Nannochloropsis* sp. is believed to be especially promising as a nutritional supplement due to the high EPA and astaxanthin levels attained in this study. Productivity, growth media requirements, environmental tolerances, and other culturing data are already established for many of these microalgae, thus progress could proceed to further clinical trialling of whole-cell or cracked-cell biomass and techno-economic assessment.
